# Schizophrenia during the COVID-19 pandemic

**DOI:** 10.1097/YCO.0000000000000702

**Published:** 2021-02-10

**Authors:** Stefano Barlati, Gabriele Nibbio, Antonio Vita

**Affiliations:** a Department of Clinical and Experimental Sciences, University of Brescia; b Department of Mental Health and Addiction Services, ASST Spedali Civili of Brescia, Brescia, Italy

**Keywords:** coronavirus, COVID-19, psychosis, SARS-CoV-2, schizophrenia, telemedicine

## Abstract

The coronavirus disease 19 (COVID-19) pandemic is having a critical impact on healthcare systems across the world, as well as on mental health in the general population; however, evidence regarding the impact of the COVID-19 pandemic on people living with schizophrenia and on the onset of psychotic symptoms is currently emerging.

**Recent findings:**

People living with schizophrenia are at an increased risk of COVID-19 and present worse COVID-19-related outcomes, including mortality. They show low levels of information and of concern regarding the possibility of contagion and infection but presented substantially stable levels of psychotic symptoms and even increased subjective well being during the pandemic. SARS-CoV-2, as well as the prolonged social isolation and the spread of misinformation, appear to be responsible in some cases for the onset of psychotic symptoms.

**Summary:**

Clinicians should inform and educate their patients on the risks related to SARS-CoV-2 infection and COVID-19 and on the precautions that they should adopt to avoid contagion. Particular attention should be devoted to maintaining the continuity of care, especially in frail patients. Telemedicine might represent a valid support, but face-to-face visits in some cases remain essential. The hypothesis of a direct role of viral infection on the onset of psychotic disorders is currently debated, as viral involvement of central nervous system appears to be rather infrequent in COVID-19.

## INTRODUCTION

### Background

The first known cases of COVID-19, the illness caused by the Severe Acute Respiratory Syndrome Coronavirus 2 (SARS-CoV-2), appeared in December 2019 [1]: since then, the outbreak has interested many countries across the globe, leading the WHO to declare it a pandemic on 11 March 2020 and many governments to orient their public health efforts in attempts to slow the spread of the disease [2]. As of January 2021, more than 79 million individuals have been infected by the virus SARS-CoV2, which has been deemed responsible of more than 1.7 million deaths worldwide [3].

Economic and ethnical disparities appear to have a relevant impact on the incidence and on the outcomes of COVID-19: marginalized groups present worst morbidity and mortality [4,5], and individuals affected by severe mental illnesses (SMIs) might be disproportionately affected by the disease [6^▪^,^7^]. In particular, people living with schizophrenia spectrum disorders (SSDs) appear to be at a high risk of infection [8] and of adverse outcomes, as they typically present poorer physical health, greater social and economic disadvantage, and, even before the outbreak, shorter life expectancy and higher excess mortality, mainly due to noncommunicable diseases [9,10].

On the contrary, the relationship between COVID-19 and mental disorders in general appears to be biunivocal, as psychiatric diagnosis might represent an independent risk factor for COVID-19, and COVID-19 survivors appear to be at increased risk of psychiatric sequelae [11^▪^] Moreover, the stress resulting from isolation and societal disruption during a pandemic and in particular during lockdown periods might have a negative effect on anxiety and depressive symptoms [12]: in this perspective, the impact of COVID-19 on the severity and even on the incidence of psychotic disorders might represent a relevant issue [13,14]. 

**Box 1:**
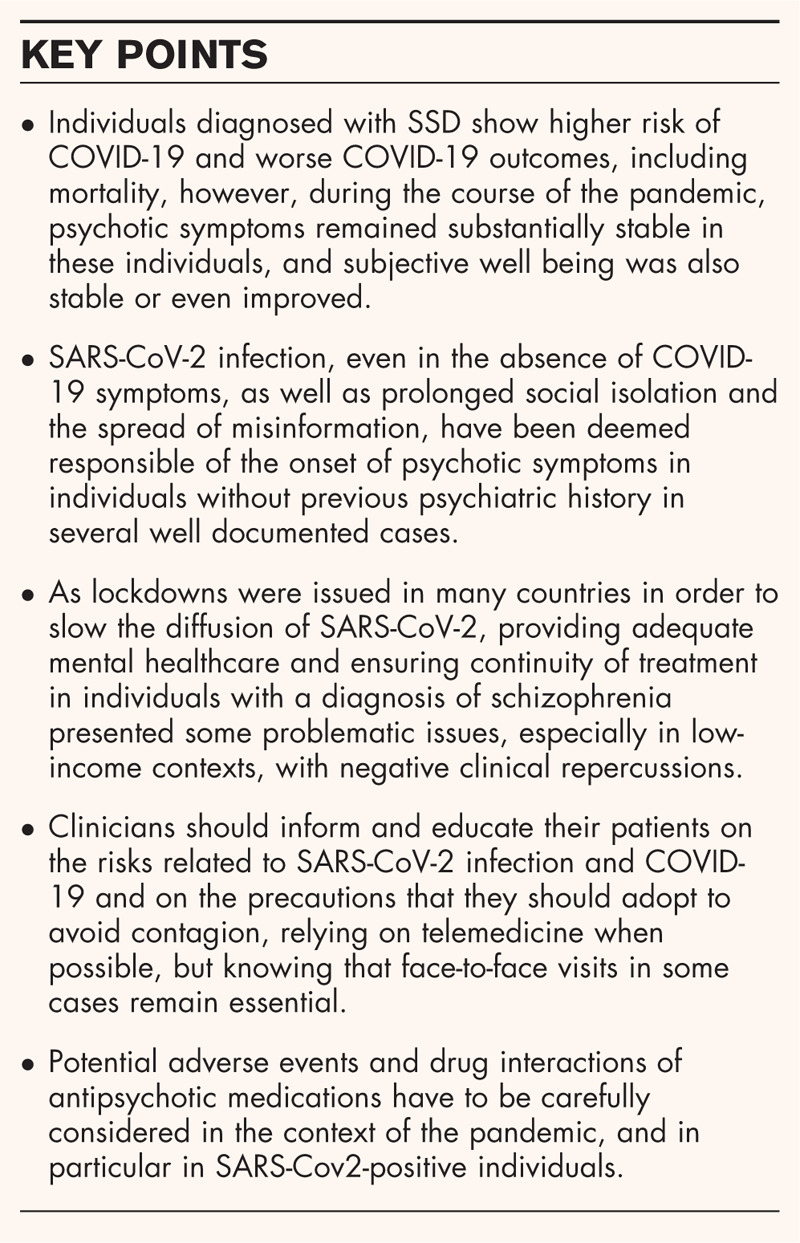
no caption available

### Aims

Although some previous work already discussed the impact of COVID-19 on the lives of people affected by psychotic disorders, providing recommendations on this topic, these are largely based on literature predating the COVID-19 outbreak, as they were issued as experts opinions in the earliest stages of the pandemic [15^▪^,^16^].

This review aims to present recent evidence on the direct and indirect effects of SARS-CoV-2 infection and of the COVID-19 pandemic on people with schizophrenia and provide a discussion on how mental health services and professionals might contribute to overcome the emerging needs related to this particular situation.

## INCIDENCE AND OUTCOMES OF COVID-19 IN PEOPLE LIVING WITH SCHIZOPHRENIA

Incidence of COVID-19 has been found to be higher in patients with a recent diagnosis of schizophrenia, compared with those without psychiatric diagnoses (adjusted odds ratio 9.89, corrected for medical comorbidities 7.34, *P* *<* 0.001 in both cases), in a study conducted on a large USA database including 61783 950 individuals, of which 15 110 were COVID-19 patients [17^▪▪^]. The same study also found a higher death rate for patients with a recent diagnosis of mental disorders (8.5 vs. 4.7%, *P* < 0.001) and a higher hospitalization rate (27.4 vs. 18.6%, *P* < 0.001).

A Korean nationwide retrospective case-control study including 219 961 individuals, 7341 of which were COVID-19 patients, also found schizophrenia, among other medical and psychiatric illnesses, to be independently associated with higher risk of COVID-19 (odds ratio range 1.61–1.72, *P* < 0.001) [18]. Psychiatric disorders, however, were not included in analyses on COVID-19 severity and outcomes.

A French study including 50 750 hospitalized individuals and focused on COVID-19 outcomes in individuals diagnosed with schizophrenia not only found an increased in-hospital mortality in this group (25.6 vs. 21.7%; adjusted odds ratio 1.30, *P* = 0.009) but also, interestingly, a decreased ICU admission rate (23.7 vs. 28.4%; adjusted odds ratio 0.75, *P* = 0.006) [19^▪^], substantially replicating the results of a study on a much smaller sample conducted by the same research group [20]. These results underline not only that individuals diagnosed with schizophrenia could suffer from more severe cases of SARS-CoV-2 infections, but also that important disparities might exist in the quality of healthcare that they receive, even in relation to COVID-19. This finding however might be influenced by the characteristics of the healthcare system and care setting: in fact, a study conducted in the UK unexpectedly found that patients with a diagnosis of psychiatric disorder were tested for COVID-19 more frequently, and globally were less likely to emerge as positive to the test. However, individuals with previous ‘psychotic experiences’ represented an exception, being positive more frequently than controls [21].

Of note, it is important to take into account that subjects with SSD often represent the majority of the residents of long-term care psychiatric rehabilitation centres, and in these facilities, the diffusion of SARS-CoV-2 can be quite fast and difficult to control: a study has found that, despite closely following the local shelter-in-place order and symptom-based testing issued in March 2020, 40 (74%) of the 50 facility residents in March and April 2020 tested positive for SARS-CoV-2, with a doubling time of 3.9 days [22].

## IMPACT OF COVID-19 PANDEMIC ON THE CLINICAL SITUATION OF PEOPLE LIVING WITH SCHIZOPHRENIA

The impact of the COVID-19 pandemic on the clinical situation of individuals diagnosed with SMI, including schizophrenia, is the focus of a growing body of scientific literature.

A study conducted in Spain on 625 patients contacted with an anonymous online questionnaire has found that, although most of the responders with a diagnosis of severe mental disorders were able to enjoy their free time during the COVID-19 pandemic lockdown period, they reported higher anxiety, stress and depression responses than the general population [23].

Another survey study conducted in India on 210 individuals diagnosed with SMI, of whom 105 had a diagnosis of SSD, found that during lockdown one out of five patients stopped their medication regimen and 30% of the sample suffered from a worsening of psychiatric symptoms. Individuals with SMI also showed low knowledge about COVID-19 symptoms and precautions to avoid infection, and three out of four did not report any worry of contracting the disease [24^▪^].

However, studies specifically taking into account people with a diagnosis of schizophrenia provide more insight on how this group of individuals fares during the pandemic.

A study conducted in Spain comparing 206 individuals with self-reported mental illnesses to 413 community controls found that those with anxiety or depressive disorders showed significantly worse psychological distress (*P* < 0.001), negative expectations about the future (*P* = 0.013) and sleep disturbances (*P* = 0.007) than those with a diagnosis of bipolar disorder or schizophrenia [25].

Similar results were found on a sample (*n* = 139) of patients in Germany, where individuals with diagnoses of affective disorders reported greater COVID-19-related stress (ANOVA *P* < 0.001 on both the adopted scales, even after controlling for potential confounding factors) than individuals with a diagnosis of SSD, leading the authors to hypothesize a potential protective role of the preoccupation with serious intrinsic issues on the stress level increase related to the pandemic situation [26^▪^].

Another German study investigating the perceived impact of the pandemic in people with various self-reported psychiatric disorders, compared with healthy controls, has found that psychosocial stress levels were higher across the whole sample, and people with mental disorders showed higher personal worries regarding COVID-19 and fear of contagion. However, although a worsening of clinical symptoms was also reported for patients with generalized anxiety disorder, illness anxiety disorders, and body dysmorphic disorder, this was not observed in subjects with SSD [27].

A recent longitudinal observational study investigated through a phone survey the changes in affective experiences and in symptoms severity between April and June 2020 in a well characterized sample of 148 USA individuals clinically diagnosed with SMI (92 diagnosed with SSD, 55 with bipolar disorder and one with major depressive disorders with psychotic features). The results of this study showed that the severity of affective experiences, of psychotic symptoms and of sleep disturbances remained substantially stable across time for individuals diagnosed with SSD (*P* > 0.05, Cohen's *d* *<* 0.20 for all measures). Although in this subsample subjective well being increased across time (*P* < 0.05, Cohen's *d* = 0.35), an increase was also observed in substance use (*P* < 0.05, Cohen's *d* = 0.13) [28^▪▪^].

Interestingly, one narrative article reported that in a specialized psychiatric rehabilitation unit, the lockdown was perceived positively by the patients, as this improved the relationship with the staff of the unit, encouraging a greater feeling of community and emphasized self and treatment awareness. Some patients also perceived the lockdown as helpful, as it brought the cessation of visits from family members with whom they had ambivalent relationships [29].

Two Chines studies compared hospitalized patients diagnosed with schizophrenia isolated for close contact with COVID-19 positive individuals or for suspect SARS-CoV-2 infection to those not isolated and, as expected, found higher levels of depression and anxiety and worse sleep disturbances in isolated patients. However, both studies found no between-groups differences in schizophrenic symptoms severity as measured by the Positive and Negative Syndrome Scale (*P* = 0.52 in one and *P* = 0.248 or *P* = 0.463 if corrected for potential confounders in the other) [30,31]. One study also compared the clinical condition of patients before and after isolation, and again, no difference was observed in schizophrenia symptoms severity (*P* = 0.36) [30].

However, SARS-CoV-2 infection could lead to symptoms relapse in clinically stable patients: the virus has been deemed responsible, in the absence of COVID-19 symptoms, of psychotic and confusional symptoms in a 65-year-old man with diagnosis of schizophrenia whose clinical picture had been stable for a very long period (20 years) [32]. His clinical situation worsened into a case of catatonia that was successfully treated with electroconvulsive therapy [33].

## IMPACT OF COVID-19 PANDEMIC ON THE INCIDENCE OF PSYCHOTIC DISORDERS

As regards the impact of COVID-19 and of the pandemic on the incidence of new cases of psychotic disorders, available evidence is composed essentially by case series and reports.

One well described case of persistent psychotic symptoms following SARS-CoV-2 infection is available. The 55-year-old female patient developed post-COVID-19 delirium, which was followed by persecutory delusions, complex visual and auditory hallucination and Capgras Syndrome for about 40 days. Although the authors did not ascertain a cause for this clinical picture, they hypothesize an inflammatory cause on the basis of the observed high levels of tumour necrosis factor (TNF)-alpha in the absence of hypoxia [34].

One interesting article reports a case of a 34-year-old man, positive for SARS-CoV-2 but with very mild symptoms (anosmia and headaches), who started showing increasingly severe disturbances and later presented a full-blown picture of schizo-affective disorder. All his laboratory and neuroimaging tests resulted substantially normal, and his symptoms subsided, albeit gradually, with relatively low dose of antipsychotic treatment [35^▪^].

Another case report describes a brief psychotic disorder in a individual with negative personal and family history of psychiatric disorders after COVID-19. Psychotic symptoms improved with treatment and subsided after the resolution of COVID-19 symptoms [36].

Other interesting reports of psychotic symptoms following SARS-CoV-2 infection in individuals without previous psychiatric history include a case series of three individuals without COVID-19 symptoms and high levels of C-reactive protein [37], another case series of three individuals whose psychotic symptoms remarkably improved with antipsychotic treatment in 2 days [38], a case of brief reactive psychosis with paranoid delusions and auditory hallucinations in a 30-year-old male individual [39], a case of affective psychosis in a 43-year-old male individual [40], a case of psychotic and confusional symptoms leading to a suicide attempt in a 34-year-old male healthcare worker [41] and a case of catatonia that responded to intravenous benzodiazepines in another 43-year-old male individual [42].

The COVID-19 pandemic could represent in some individuals a precipitating factor even in the absence of SARS-CoV-2 infection: prolonged social isolation, fear of contagion and the uncontrolled spread, especially online, of pandemic-related conspiracy theories and delusion-like beliefs, dubbed by some authors as the ‘infodemic’, might be linked not only to high levels of anxiety, depressive symptoms and sleep disturbances, but also to psychotic symptoms.

In fact, a study conducted in Spain on 174 individuals recruited in the general population found that perceptual disturbances, subclinical psychotic symptoms and pseudoscientific beliefs increased across the total social quarantine imposed to control the viral spread [43].

An interesting case series reported that three individuals with negative psychiatric history (a 23-year-old man, a 30-year-old woman and a 37-year-old man), all testing negative to SARS-CoV-2, developed delusions specifically related to the COVID-19 pandemic: in each of these cases, the psychotic symptoms led to a first psychiatric hospitalization [44].

Another case series reports of six first-episode psychoses, confirmed later as brief psychotic disorder or acute transient psychotic disorder, of individuals with negative psychiatric history and advanced age. All patients tested negative for SARS-CoV-2 and had religious-spiritual delusions and hallucinations; half of the individuals had somatic delusions of being infected. All patients required hospitalization but were rapidly discharged with remission of symptoms and good insight after relatively low-dose antipsychotic treatment [45].

## IMPACT OF COVID-19 PANDEMIC ON TREATMENT OF SCHIZOPHRENIA

The outbreak of COVID-19 represents a relevant issue as regards the safety profile of psychotropic medications, as some relevant drug-drug interactions might be present with drugs used for COVID-19 treatment, and some psychotropic medications might produce adverse effect that could be seriously dangerous in COVID-19 patients. A systematic review providing important experts recommendations has been conducted on this topic [46^▪▪^]: main recommendations regarding antipsychotic drug use are to check for potential drug interactions and avoid dangerous associations, carefully assess respiratory function, avoiding molecules that could cause respiratory depression particularly in the long term, and carefully monitor cardiac parameters including QTc, the risk for secondary infections and the risk of thromboembolism.

Individuals treated with long-acting injectable (LAI) antipsychotics should continue receiving this form of treatment, as it has a considerable positive impact on relapse prevention, even if in-person visits, which are required for administering the injection, might represent a potential source of contagion as they require close physical proximity [15^▪^,^47^]. An interesting retrospective observational study conducted in Romania shows an important reduction of LAI antipsychotics prescriptions during the pandemic in favour of oral formulations [48]. The authors comment that this could be due to the reduction of in-person visits, to their cost and to delays in pharmacies supply, and might lead to a higher incidence of relapses in the near future. This represents a relevant issue, that could be particularly problematic in less wealthy countries. In fact, a study reports that in Pittsburgh, USA no similar decrease in the prescription of LAI was observed. However, the authors also report that their region was not a hot-spot of SARS-CoV-2 transmission [49].

Clozapine treatment in particular represents an issue, both because it increases susceptibility to infections and because monitoring of absolute white cell blood count might become difficult to implement in accordance with the restrictions adopted in various countries to slow the spread of contagion. An expert consensus has considered this issue and recommended that in stable patients the frequency of neutrophils count can be reduced to once every 3 months; in patients with any symptoms of infection, urgent physician assessment and complete blood count should be immediately carried out; in patients presenting fever or flu-like symptoms, clozapine dose should be lowered by as much as half [50]. Some authors have argued that this reduction might not be enough in patients who are probably facing COVID-19 symptoms, and recommend lowering the dose to a third or stopping it completely [51]; other suggest to provide Vitamin D supplementation in all patients treated with clozapine to increase protection against respiratory infections [52]. The use of clozapine has also been associated with increased risk of COVID-19 in a retrospective cohort study conducted in the UK including 6309 participants [53].

On the contrary, an interesting series composed of seven cases from the UK reports that, with some caution and clinical attention, clozapine can be safely used in individuals affected by COVID-19, effectively preventing relapses of psychotic symptoms, after ruling out that clozapine might be responsible of some of the observed symptoms [54]. Telephonic monitoring, including telematic transmission of absolute neutrophils count, might also represent a possible and viable solution to maintain treatment continuity in individual treated with clozapine [55].

Telemedicine-videoconferencing and e-mental health represent important tools to keep continuity of care in individuals diagnosed with SMI without increasing the risk of viral spread with in-person visits [47,56].

Some studies have shown that enhancing evidence-based interventions, such as assertive community treatment, with mobile-based interventions had a positive effect on clinical outcomes in individuals with SMI including schizophrenia [57], or that digital health interventions designed to improve relapse prevention in individuals with first episode psychosis are deemed feasible and appreciated by both patients and clinicians [58]. However, the actual effectiveness of such interventions is not yet supported by solid evidence, and some authors consider face-to-face interventions essential in the psychiatric care of SSD, especially for new cases with suspected psychosis or for individuals with technological delusions or phobias [59].

## DISCUSSION

Current and solid evidence attests that people diagnosed with schizophrenia, compared with the general population, show higher COVID-19-related mortality and increased risk of contracting COVID-19 [17^▪▪^].

The first might be due to the higher frequency of comorbid medical conditions [10] and, to a certain extent, to discrepancies in the quality of care, which result from stigma [19^▪^,^60^]; the latter might probably be related to the low level of information regarding COVID-19 and of concern regarding the possibility of infection that have both been observed in individuals diagnosed with SMI [24^▪^].

Both the increased risk of COVID-19 and the higher mortality could be also related to the unhealthy lifestyle that is frequently observed in subjects living with schizophrenia; in particular, the habit of tobacco smoking could have a negative impact on prognosis in case of SARS-CoV-2 infection.

The low level of information regarding COVID-19, in turn, could be determined by various factors, such as the lower level of education or the cognitive deficits; the low level of concern may be linked not only to the lack of information, but also to cognitive deficits and autistic features, such as preoccupation with intrinsic issues, that represent central elements of the disorder [61].

The low level of awareness and concern regarding the virus and the pandemic, however, might be associated with the substantially stable psychotic symptoms and stable or even improved subjective well being, which have been observed in some studies [28^▪▪^]. In this context, some authors also hypothesized a protective role of intrinsic preoccupation in patients with SSD [26^▪^].

**FIGURE 1:**
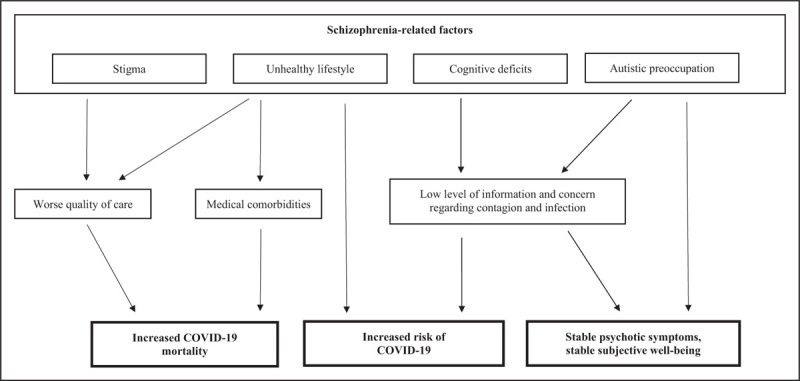
The impact of COVID-19 pandemic on people living with schizophrenia: risk factors and clinical consequences. Schizophrenia-related features, including some core elements of the disorder, might represent direct and indirect risk factors related to COVID-19 and worse COVID-19 outcomes, including mortality, and have relevant clinical consequences in the context of the pandemic.

These correlations (see Fig. 1), however, at present remain hypothetical and should be confirmed with more evidence derived from methodologically appropriate studies.

COVID-19 has been deemed responsible, in some cases, of the onset of psychotic symptoms and disorders or of the exacerbation of psychotic symptoms in subjects with a stable clinical picture [32,34,35^▪^,^36^]. Some of these cases however might indicate a direct neurological involvement, since they had a clinical presentation including atypical and confusional symptoms, closer to delirium than to SSD [32,41].

Although the hypothesis of a role of viral or immuno-mediated mechanisms in the pathophysiology of psychotic disorders is of great potential interest [35^▪^], the current literature regarding this aspect of SARS-CoV-2 is still anecdotal and this interesting issue remains debated [62,63].

Of note, the potential impact of prenatal and perinatal SARS-CoV-2 infection on individual's neurodevelopment represents an issue requiring close scientific and clinical attention in the forthcoming years [64–66].

Despite in the last year a substantial number of papers regarding COVID-19 and psychiatric disorders, including schizophrenia, have been published, the vast majority of this wealth of scientific literature is composed by studies with relevant intrinsic methodological limitations, case series and case reports, or experts’ opinions and consensuses. In this context, beyond some recent and noteworthy exceptions, there is the need to acquire a more robust body of scientific evidence on this topic.

## CONCLUSION AND FUTURE DIRECTIONS

Clinicians should inform and educate their patients on the risks related to SARS-CoV-2 infection and COVID-19 and on the precautions that they should adopt to avoid or reduce the risk of contagion. Particular attention should be devoted to maintaining the continuity of care, especially in frail patients. Telemedicine might represent a valid support, but face-to-face visits in some cases remain essential, and have to be conducted with all the necessary precautions to avoid infection for both patients and clinicians. More solid evidence should be gathered on the effects of COVID-19 and of the pandemic in people living with schizophrenia, on the potential risk of psychosis related to SARS-CoV-2 infection, as well as on the safety and the possible interactions of antipsychotic medications and drugs used to treat COVID-19.

## Acknowledgements

*None.*

### Financial support and sponsorship

*None.*

### Conflicts of interest

*There are no conflicts of interest.*

## REFERENCES AND RECOMMENDED READING

Papers of particular interest, published within the annual period of review, have been highlighted as:

▪ of special interest
▪▪ of outstanding interest
